# Supplementary material for: The Antibacterial Effect of PEGylated Carbosilane Dendrimers on P. aeruginosa Alone and in Combination with Phage-Derived Endolysin
Source: Int J Mol Sci. 2022 Feb 7;23(3):1873. doi: 10.3390/ijms23031873 (PMC8836974; doi:10.3390/ijms23031873)
Supplement: Supplementary file 1 [file ijms-23-01873-s001.zip › ijms-1563671-supplementary.pdf]

For supporting information

The antibacterial effect of PEGylated carbosilane dendrimers on *P. aeruginosa* alone and in the combination with phage-derived endolysin

Sara Quintana-Sanchez[1,2,3], Natalia Gómez-Casanova[4], Javier Sánchez-Nieves[1,2,3,*], Rafael Gómez[1,2,3], Jarosław Rachuna[5], Sławomir Wąsik[6], Jacek Semaniak[6], Barbara Maciejewska[7], Zuzanna Drulis-Kawa[7], Karol Ciepluch[5], Francisco Javier de la Mata[1,2,3], Michał Arabski[5,*]

1 Department of Organic and Inorganic Chemistry, Research Institute in Chemistry "Andrés M. del Río" (IQAR), University of Alcalá, 28871 Alcalá de Henares, Spain; sara.quintana@edu.uah.es (S.Q.-S.); rafael.gomez@uah.es (R.G.); javier.delamata@uah.es (F.J.d.l.M.)
2 Networking Research Center for Bioengineering, Biomaterials and Nanomedicine (CIBER-BBN), 28029 Madrid, Spain
3 Ramón y Cajal Institute of Health Research, IRYCIS, 28034 Madrid, Spain;
4 Department of Biomedicine and Biotechnology, Faculty of Pharmacy, University of Alcalá, 28871 Alcalá de Henares, Spain; natalia.gomezc@uah.es
5 Division of Medical Biology, Jan Kochanowski University, 25-406 Kielce, Poland; jaroslaw.rachuna@gmail.com (J.R.); kciepluch@ujk.edu.pl (K.C.)
6 Institute of Physics, Jan Kochanowski University, 25-406 Kielce, Poland; slawomir.wasik@ujk.edu.pl (S.W.); jacek.semaniak@ujk.edu.pl (J.S.)
7 Department of Pathogen Biology and Immunology, University of Wroclaw, 51-148 Wroclaw, Poland; barbara.maciejewska@uwr.edu.pl (B.M.); zuzanna.drulis-kawa@uwr.edu.pl (Z.D.-K.)
* Correspondence: javier.sancheznieves@uah.es (J.S.-N.); arabski@ujk.edu.pl (M.A.)

4.1. *Synthesis of cationic CBS dendrimers.*

$G_0Si(SiMe_2V)_4$ (1):

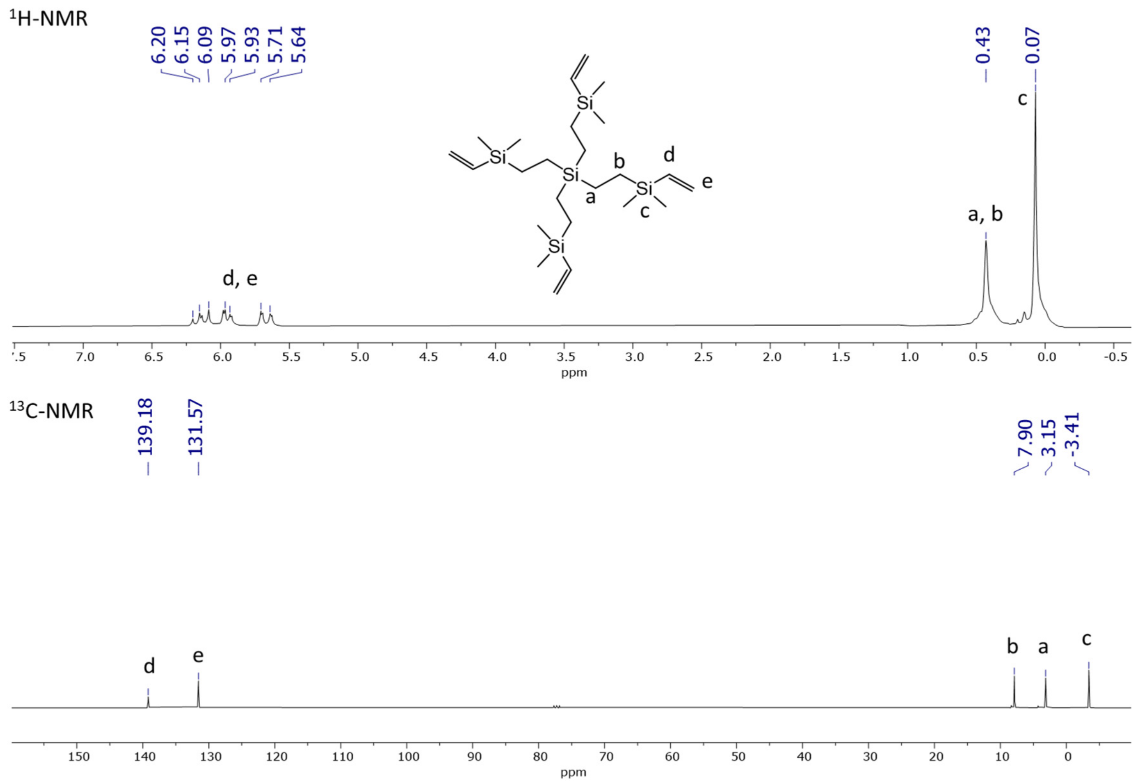

Figure S1. ^{1}H and ^{13}C-NMR of compound **1** ($CDCl_3$).

G$_0$Si(SiMe$_2$-NMe$_2$HCl)$_4$ (2):

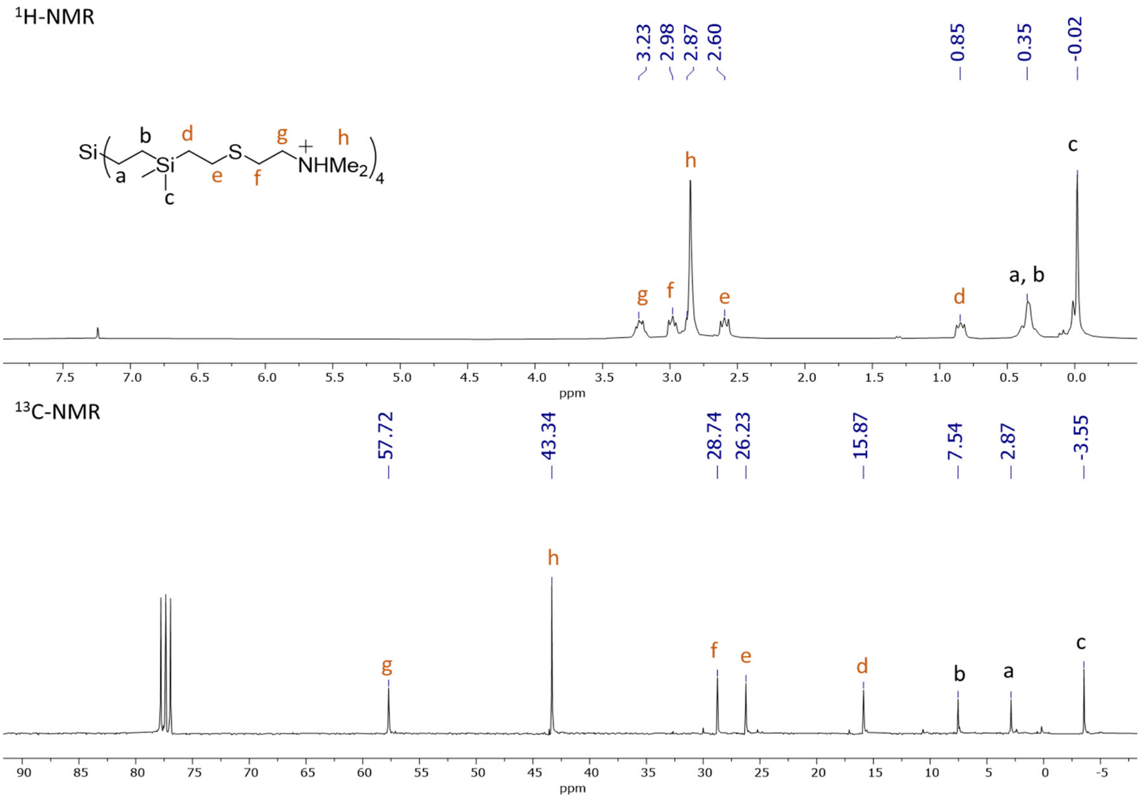

Figure S2. ^{1}H and ^{13}C-NMR of compound **2** (CD_3OD).

G$_0$Si(SiMe$_2$-NMe$_2$)$_4$ (3):

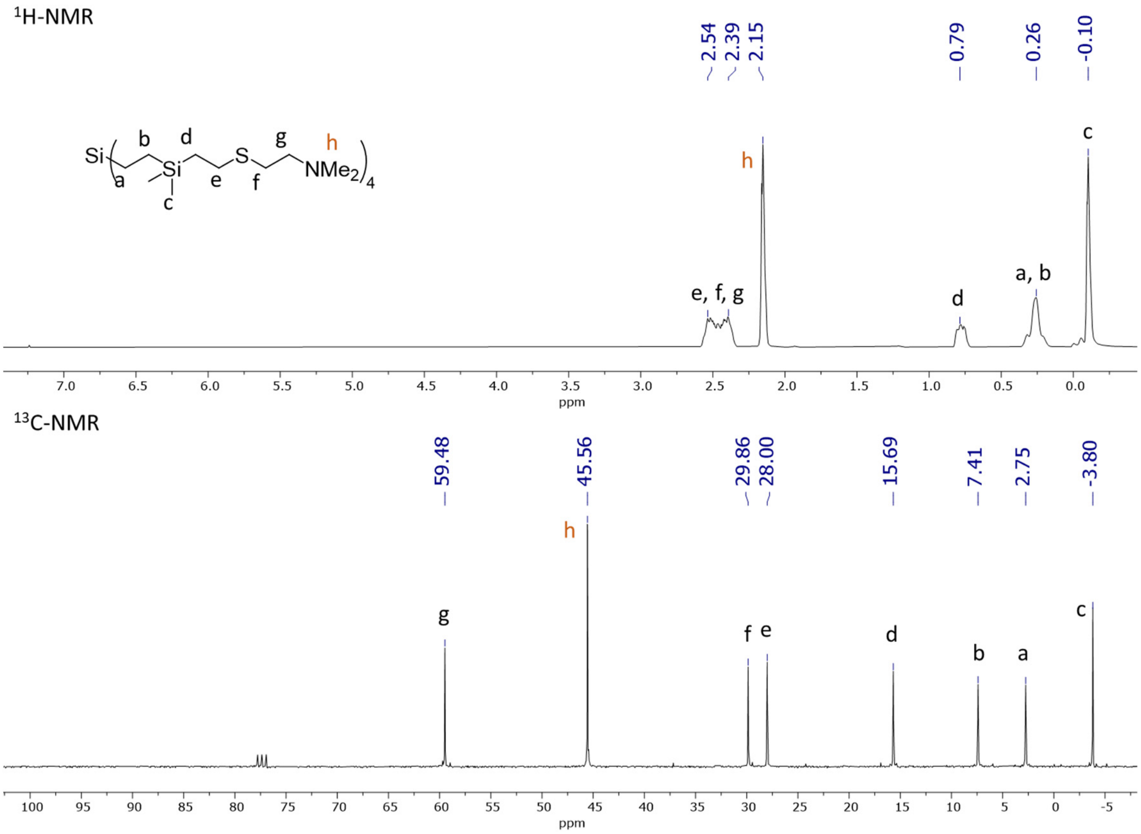

Figure S3. ^{1}H and ^{13}C-NMR of compound **3** ($CDCl_3$).

$G_0Si(SiMe_2\text{-}NMe_3Cl)_4$ (4):

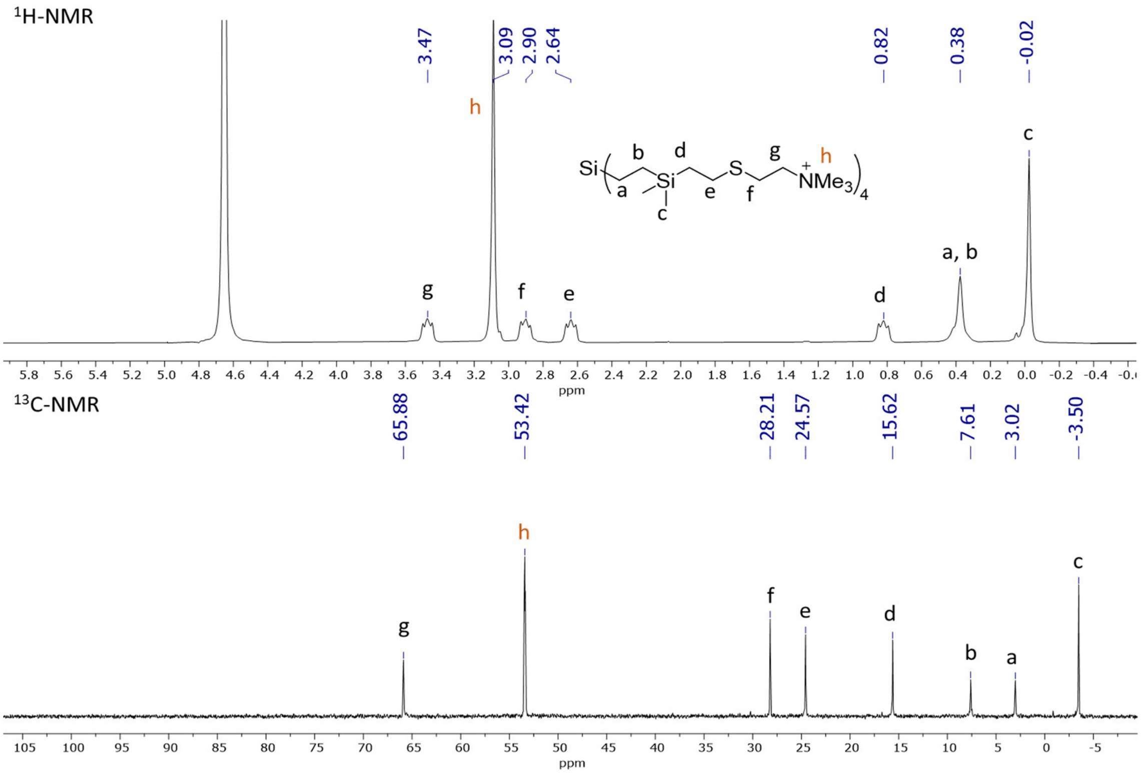

Figure S4. ^{1}H and ^{13}C-NMR of compound **4** (D_2O).

$G_0Si(SiMe_2\text{-}PEG800)(SiMe_2\text{-}NMe_2HCl)_3$ (5):

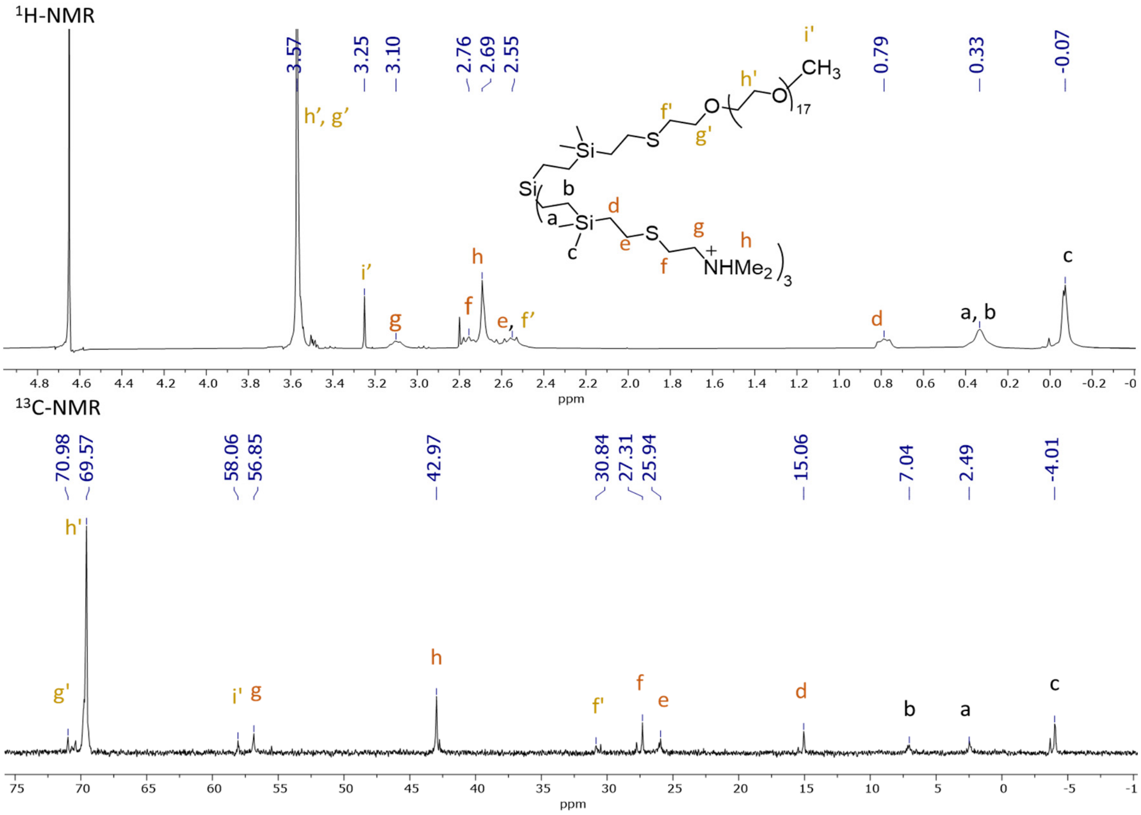

Figure S5: ^{1}H and ^{13}C-NMR of compound **5** (CD_3OD).

$G_0Si(SiMe_2\text{-}PEG800)(SiMe_2\text{-}NMe_2)_3$ (6):

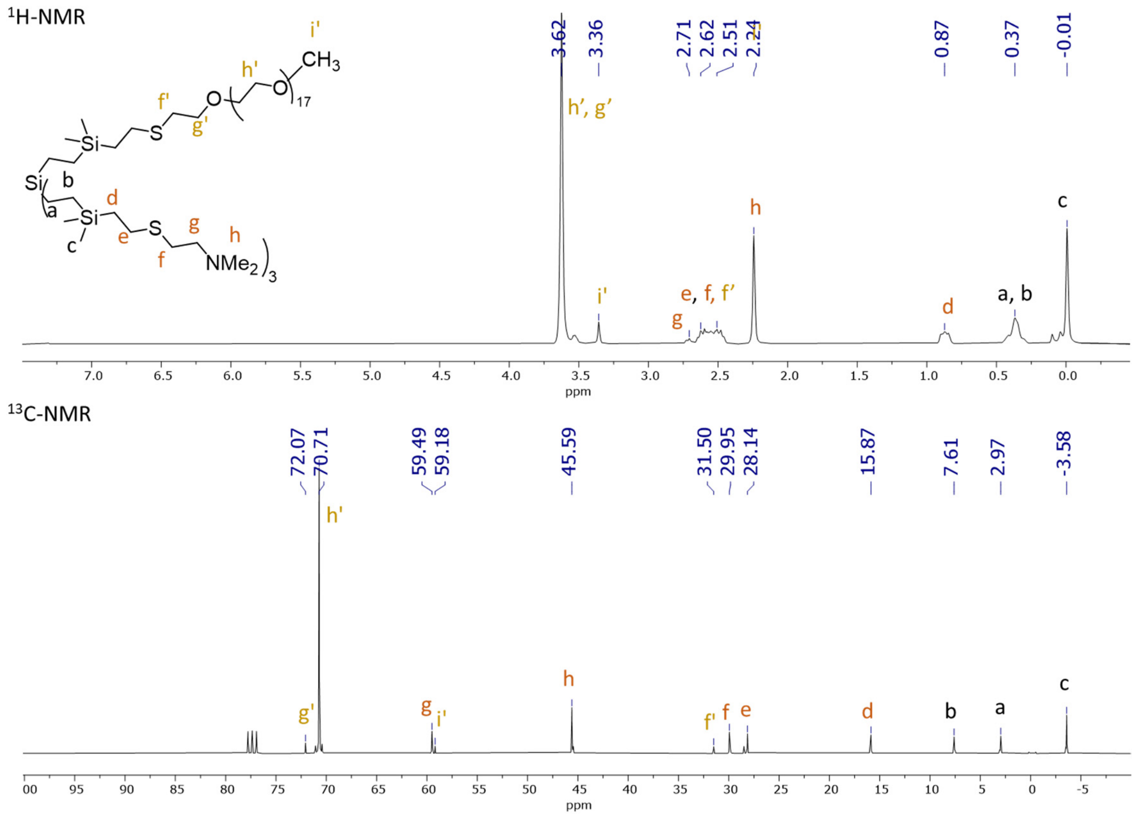

Figure S6. ^{1}H and ^{13}C-NMR of compound **6** ($CDCl_3$).

$G_0Si(SiMe_2\text{-}PEG800)(SiMe_2\text{-}NMe_3Cl)_3$ (7):

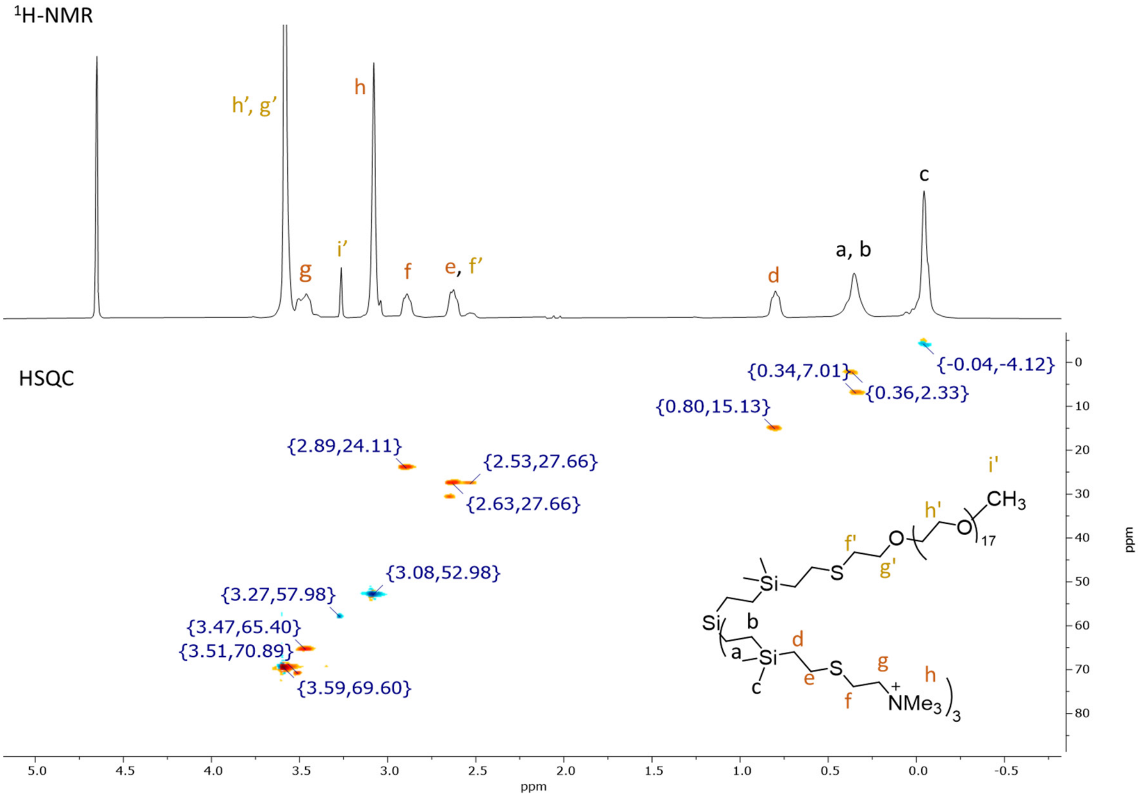

Figure S7. ^{1}H and HSQC of compound 7 (D_2O).

G$_0$Si(SiMe$_2$-PEG2K)(SiMe$_2$-NMe$_2$HCl)$_3$ (8):

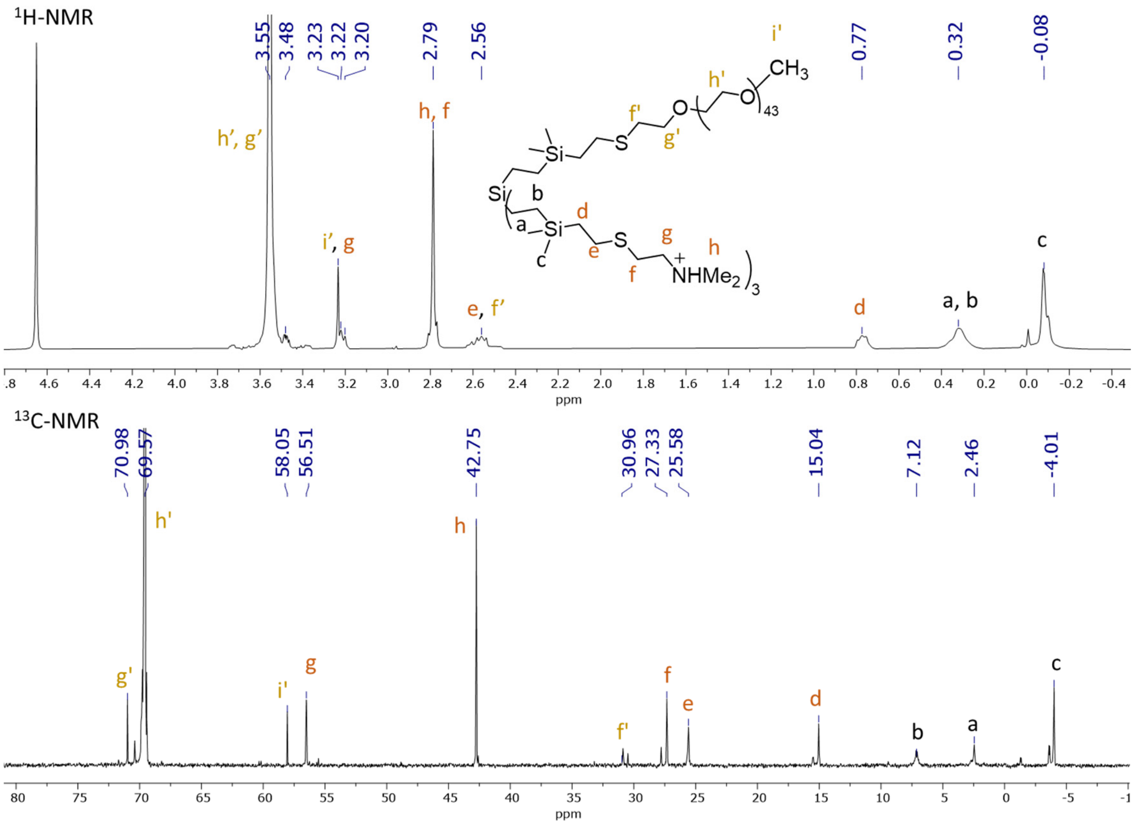

Figure S8: ^{1}H and ^{13}C-NMR of compound **8** (CD_3OD).

G$_0$Si(SiMe$_2$-PEG2K)(SiMe$_2$-NMe$_2$)$_3$ (9):

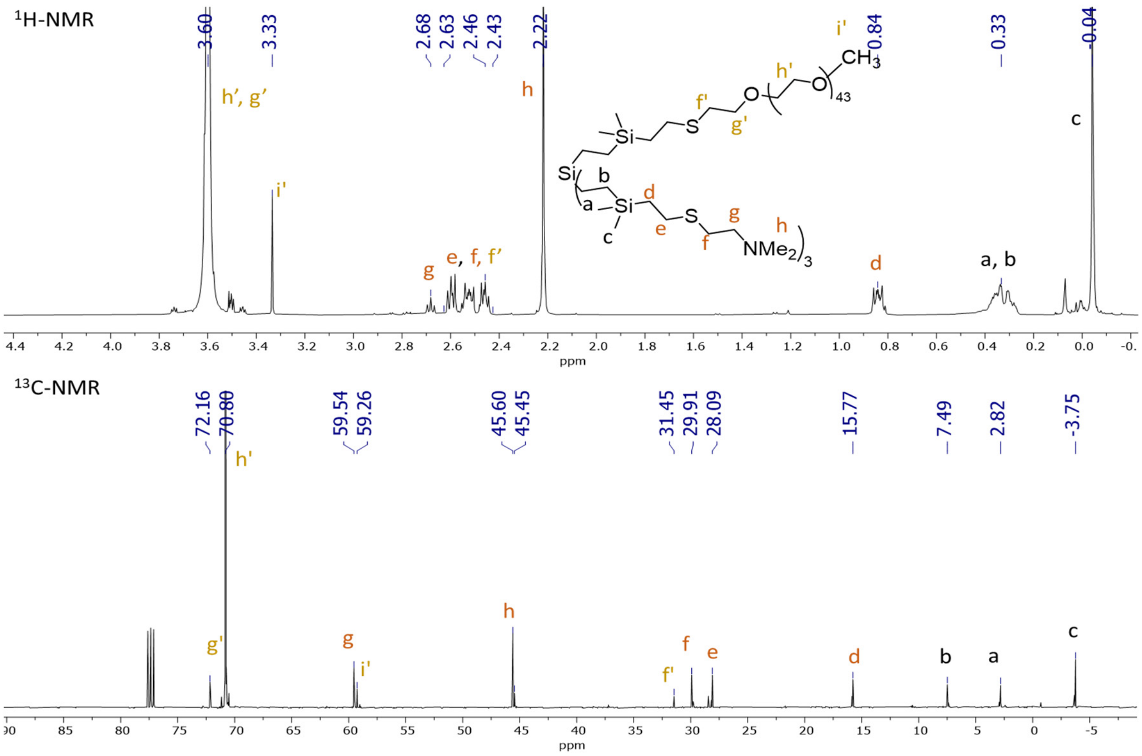

Figure S9. ^{1}H and ^{13}C-NMR of compound **9** ($CDCl_3$).

G$_0$Si(SiMe$_2$-PEG2K)(SiMe$_2$-NMe$_3$Cl)$_3$ (10):

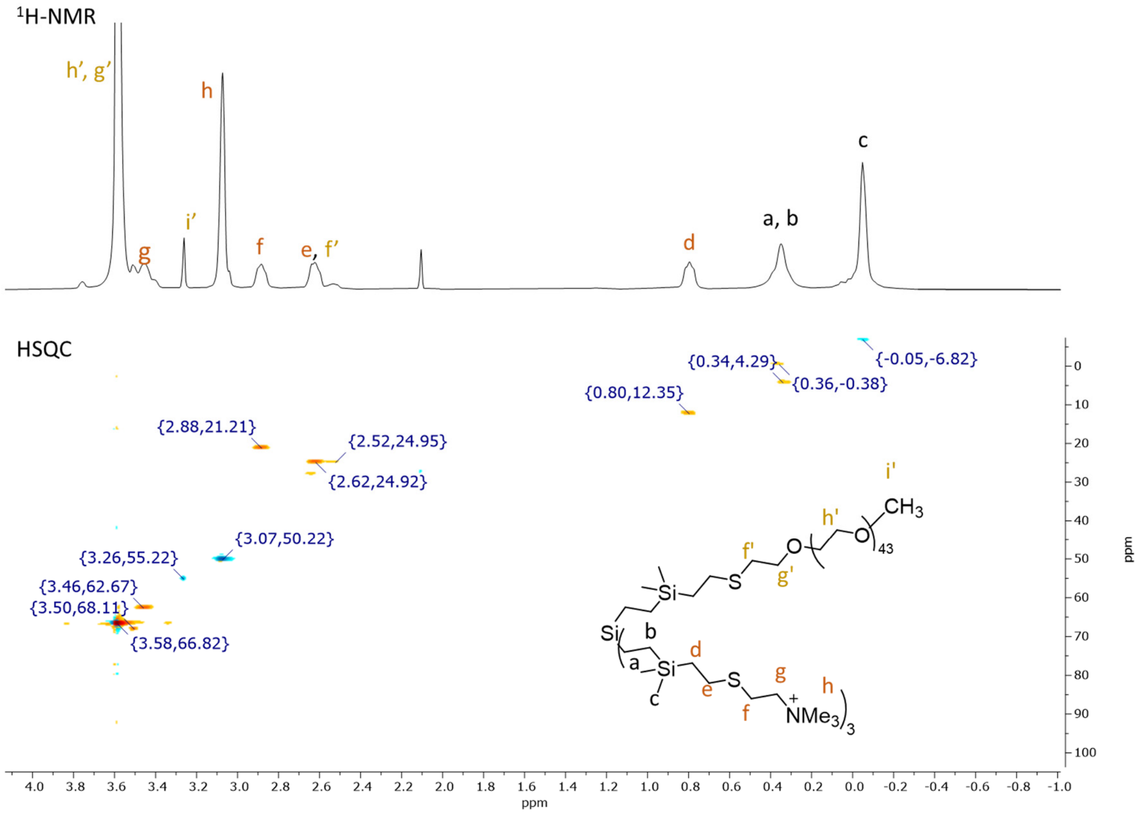

Figure S10. ^{1}H and HSQC of compound 10 (D_2O)

Table S1. Relationship between antibacterial concentration in mg/l and μM used in this work.

ppm → (mg/L)	**4** (μM)	**7** (μM)	**10** (μM)
1024	928	586	347
512	464	293	174
256	232	146	87
128	116	73	43
64	58	37	22
32	29	18	11
16	14.5	9.1	5.4
8	7.25	4.6	2.7
4	3.6	2.3	1.4
2	1.8	1.1	0.7
1	0.9	0.6	0.3
0.5	0.4	0.3	0.2
0.25	0.2	0.1	0.08
0.125	0.1	0.07	0.04

Table S2. Antibacterial activity of cationic CBS dendrimer (**4**) and with pegylated cationic CBS dendrimer PEG-800 (**7**) or PEG-2000 (**10**) against *P. aeruginosa* planktonic cells and biofilm. Data are in μM.

**Dendrimer (compound no.)**	**Planktonic cells**		**Biofilm**			
			**Preventing biofilm formation**		**Removing biofilm**	
	**MIC**	**MBC***	**MBIC**	**MBBC***	**MBDC**	**MBEC***
**4**	29	29	58	116	116	464
**7**	146	146	293	586	586	>586
**10**	87	87	>347	>347	>347	>347

*Drop Plate Method. Planktonic cells: Minimum Inhibitory Concentration (MIC) and Minimum Bactericide Concentration (MBC). Biofilm: Minimum Biofilm Inhibitory Concentration (MBIC), Minimum Bactericidal Concentration in Biofilm (MBBC), Minimum Biofilm Damaging Concentration (MBDC) and Minimum Biofilm Eradication Concentration (MBEC).
